# Impact of dose reducing software on patient and staff temple dose during fluoroscopically guided pacemaker insertion, closure devices implantation and coronary angiography procedures

**DOI:** 10.1007/s13246-022-01126-2

**Published:** 2022-05-09

**Authors:** Kelly S. Wilson-Stewart, Davide Fontanarosa, Eva Malacova, Jamie V. Trapp

**Affiliations:** 1 School of Chemistry and Physics, Faulty of Science, Queensland University of Technology, 2 George Street, Brisbane, QLD 4000 Australia; 2 Cardiovascular Suites, Greenslopes Private Hospital, Ramsay Health Care, Newdegate Street, Greenslopes, Brisbane, QLD 4120 Australia; 3 School of Clinical Sciences, Faculty of Health, Queensland University of Technology, 2 George Street, Brisbane, QLD 4000 Australia; 4 Centre for Biomedical Technologies (CBT), Queensland University of Technology, Brisbane, QLD 4000 Australia; 5 QMIR Berghofer Medical Research Institute, 300 Herston Road, Herston, QLD 4006 Australia

**Keywords:** Noise reduction, Occupational exposure, Cardiac closure devices, Nursing dose

## Abstract

The aim of this study is to investigate the effectiveness of dose reducing software (ClarityIQ) on patient and staff dose during fluoroscopically guided cardiac procedures. Dose measurements were collected in a room without dose reducing software (n = 157) and compared with similar procedures performed in two rooms with the software (n = 1141). Procedures included diagnostic coronary angiography, percutaneous coronary intervention, deployment of cardiac closure devices (for occlusion of atrial septal defect, patent foramen ovale, and atrial appendage) and insertion of permanent pacemakers. The dose reducing software was found to be effective in reducing patient and staff dose by approximately 50%. This study has added to the limited literature reporting on the capability of dose reducing software to decrease radiation exposure during the implantation of cardiac closure devices, as well as demonstrating a reduction in dose to the cardiologist and nursing staff. Administrators should ensure timely upgrades to angiographic equipment to safeguard patients and staff against the potentially adverse effects of radiation exposure. Regardless of the use of dose reducing software, the mean occupational dose during closure devices was in descending order scout > scrub > cardiologist. Scrub nurse dose was found to be higher than the cardiologist during closure devices (0.98/0.26 μSv) and diagnostic coronary angiograms (1.51/0.82 μSv). Nursing staff should be aware that their levels of radiation dose during some cardiac procedures may come close to or even exceed that of the cardiologist.

## Introduction

The number and complexity of fluoroscopically guided procedures continue to grow, as does the concern over elevated levels of radiation dose. The rising number of high dose imaging investigations increases the stochastic risk of oncogenesis with lengthy procedures also resulting in patient skin erythema [1]. Radiation exposure in the catheterization laboratory (cath lab) is notably higher than that of other fluoroscopic departments [2], and increased rates of subcapsular cataracts have been reported in cath lab staff [3]. There is also a suspected causal relationship between occupational exposure and the incidence of breast, brain, and skin cancer [4, 5].

Manufacturers of angiographic equipment are mindful of the growing procedural complexity and the associated increase in exposure levels and are attempting to mitigate this through the development of dose reduction technology. ClarityIQ (Philips Healthcare, Netherlands) is a noise reduction software with an optimized image processing chain that employs algorithms for real-time motion compensation. In addition, it utilizes temporal averaging of consecutive images to allow for temporal noise reduction, spatial filtering for spatial noise reduction, as well as image enhancement tools such as edge and contrast enhancement [6–8]. Algorithms optimize image quality for specific clinical applications [9]. It has been reported that the use of ClarityIQ results in a reduction in dose rate, allowing additional filtration to be used to improve photon penetration.[10].

Previous studies have investigated the effectiveness of this technology in reducing patient dose during adult cardiac procedures (Table 1), with almost universal reporting on a decrease in patient dose area product (DAP), with no loss in perceived image quality. However, there are differing results on the effect of dose reducing software (DRS) on occupational dose. Some authors report a reduction in dose to the staff during (non-cardiac) endovascular procedures [10–12], while others indicate that DRS results in an increase in scattered radiation and consequently causing an increase in occupational dose to staff [13, 14]. There are a number of procedures for which the effects of DRS have not been well studied [15], including the insertion of closure devices for the treatment of pathologies such as patent foramen ovale (PFO), atrial septal defect (ASD), as well as atrial appendage closures in the clinical setting.

**Table 1:** Previous studies investigating the effect of ClarityIQ software on patient and staff exposure during coronary angiography and the insertion of permanent pacemakers and closure devices

	Year	Procedures	Total number of procedures	DRS	DAP	% reduction	AK	% reduction	Staff	% reduction	Image quality assessment
Eloot et al. [12]	2015	DCA	70	Clarity	√	75	–	–	–	–	Similar
ten Cate et al. [13]	2015	DCA	39	Clarity	√	53	√	53	–	–	Similar
Maccagni et al. [14]	2018	CTO	60		√	79	√	73	–	–	Similar
			127	Clarity							
Kastrati et al. [15]	2016	DCA	397		√		–	–	–	–	Similar
		PCI	208								
		DCA	435	Clarity		65					
		PCI	260			69					
Studzińska et al. [16]	2017	ACD	*	Clarity	–	49–86	–	–	–	–	–
Balter [17]	2017	DCA	3947	Clarity	√	34–44	√	45–65	–	–	–
		CTO	276			23–28		43–48			
Gunja et al. [18]	2017	DCA	145		√		–	–	–	–	Similar
		PCI	55								
		DCA	143	Clarity		45–60					
		PCI	57								
Hoffmann et al. [11]	2017	Pacemakers, ICD, CRT	132		√	60	–	–	–	–	Similar
			147	Clarity							
Sharma et al. [19]	2017	ACD	9	Clarity			√	69	–	–	–
		Ablation procedures	35					95			
		PPM/ICD	46					96			
Memon et al. [20]	2018	DCA, PCI, peripheral angiography, RHC, PPM +	467		√	42	–	–	–	–	–
			688	Clarity							
Busse et al. [21]	2018	CTO	98		√	34	√	30	–	–	–
			98	Clarity							
Salinas et al. [22]	2020	CTO	60		√	36	√	47	Operator (77 procedures)	0	–
			56								
Kraemer et al. [23]	2021	DCA	119		√	56					
			119	Clarity							

*ACD* atrial closure device, *AK* air kerma, *CRT* cardiac resynchronization therapy, *CTO* chronic total occlusion, *DAP* dose area product, *DCA* diagnostic angiography, *DRS* dose reducing software, *ICD* implantable cardioverter defibrillator, *PCI* percutaneous coronary angiography, *PPM* permanent pacemaker, *RHC* right heart catheterization

^*^Phantom study—assessed organ dose; ^Clarity—ClarityIQ technology (Philips Healthcare, Netherlands); + additional procedures also included in sample

Furthermore, there are very few studies investigating the effect of DRS on staff, especially nursing staff. This study aims to quantify the impact of ClarityIQ DRS on patient, cardiologist, scrub and scout nurse dose during the implantation of permanent pacemakers, cardiac closure devices and interventional and diagnostic coronary angiography. The levels of occupational and patient dose will be compared for a room without the software upgrade, to rooms equipped with the dose reducing software.

## Materials and methods

This single-center study was conducted between February 2017 and August 2019 at a large Australian tertiary hospital in Brisbane. The occupational dose to the 24 cardiologists (1202 cases), 32 scrub nurses (1101 cases) and 35 scout nurses (737 cases) was measured by Philips DoseAware (Philips, Netherlands) dosimeters worn near their temple. DoseAware is an active personal dosimeter that has previously been reported to respond satisfactorily in realistic scatter fields such as those in interventional cardiology [16] and has been verified when compared to thermoluminescent dosimetry [17]. The angular response stated for DoseAware is ± 5% within ± 5°, ± 30% within ± 50° and + 200%/ − 100% within ± 90° for an energy range up to 120 keV [18], which is within the International Electrotechnical Commission recommendations of an angular response from 0° to 60° for the energy range 20–100 keV [19].

Staff dosimeters were attached as close as practicable to the temple nearest the x-ray tube as this has been shown to receive significantly higher levels of scatter dose than the side of the head furthest away from the x-ray source [20]. All in-room staff wore lead garments including thyroid shields, with scrub staff also wearing shin protection. The cardiologists typically wore lead goggles, with the majority of scrub nurses wearing lead skull caps in addition to goggles. DoseAware badges were worn external to any protective equipment.

All procedures were performed in one of three dedicated angiographic suites using Philips Allura Xper equipment (Philips Healthcare, Netherlands). Room 1 (R1) and Room 2 (R2) had a 10-inch square detector with nominal focus spot sizes of 0.4/0.7 mm. Room 3 (R3) had a 19-inch rectangular detector with 0.5/0.8 mm focus spot sizes, allowing better heat dissipation. Both systems had the same inherent filtration and utilised identical generator systems [21].

**Fig. 1:**
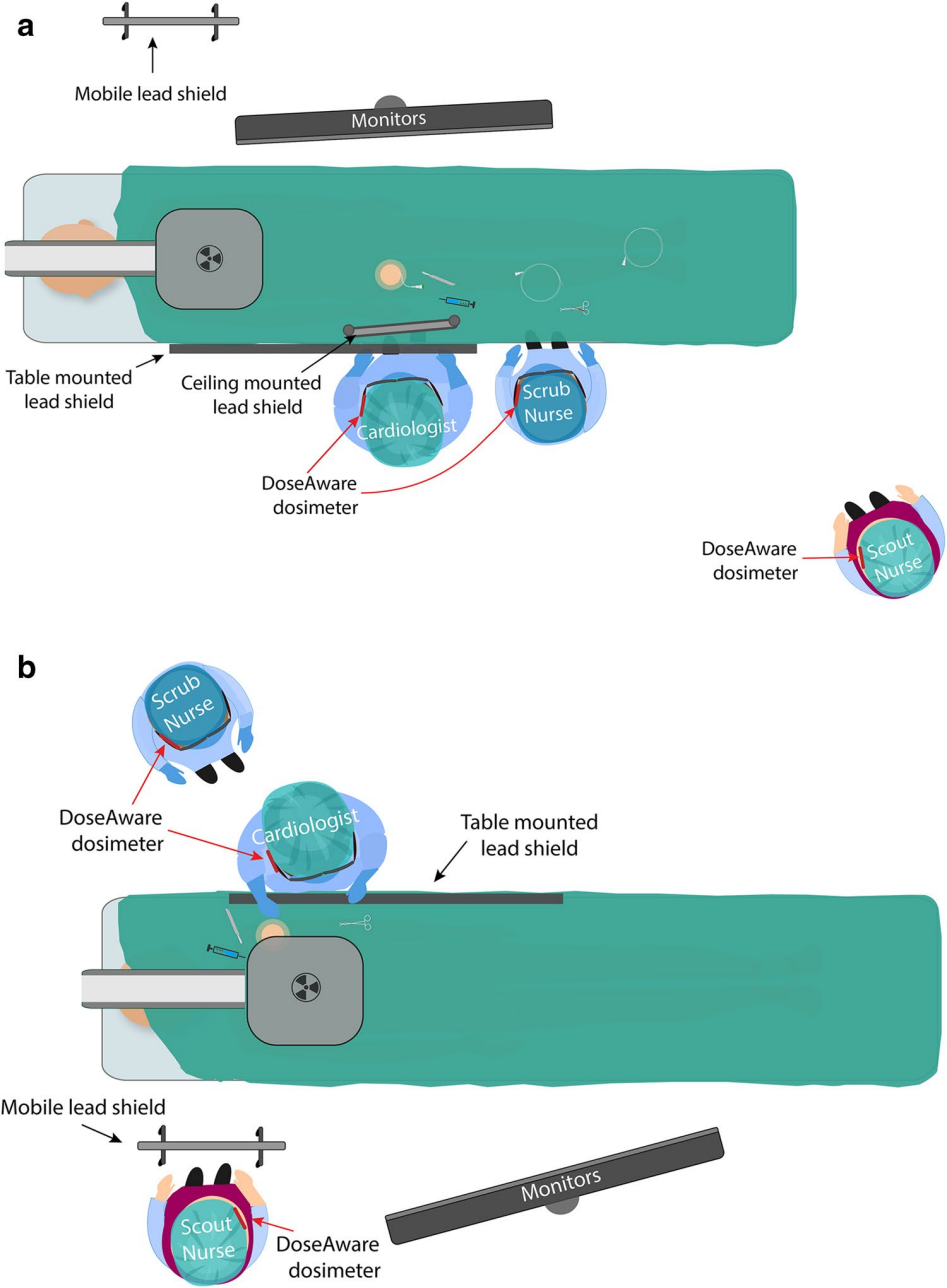
Typical equipment and staff arrangement during **a** coronary angiography and intervention and closure device insertion and **b** permanent pacemaker implantation

R1 did not have DRS installed. R2 and R3 had the Clarity IQ DRS installed and were thus more frequently utilized for coronary angiography procedures. R1 was predominantly used for insertion of permanent pacemakers, typically with low-dose cine and fluoroscopy frame rates of 7.5 frames per second (fps). The default setting during angiography and closure devices was 15fps. Typical shielding arrangement and staff positioning is demonstrated in Fig. 1.

Patient dose parameters of DAP (Gy·cm2) and air kerma (AK) (Gy) were collected for each case (n = 1537). The accuracy of the DAP meters was tested twice yearly and confirmed using external dosimeters. Procedures were categorized into diagnostic coronary angiography, percutaneous coronary intervention, implantation of PFO, ASD and atrial appendage closure devices, and insertion of permanent pacemakers. Due to the reports of high levels of radiation exposure during biventricular (BiV) implantable cardioverter defibrillator procedures, this was considered separately.

### Ethics approval

Institutional ethics approval was granted by the Ramsay Human Research Ethics Committee (Protocol number–16/67). Informed, written consent was obtained from staff participants, and since all identifying information was removed prior to analysis, patient consent was deemed unnecessary by the ethics committee. All components of this study were conducted in accordance with the Declaration of Helsinki. The authors affirm that consent has been received to publish images of staff participants.

### Statistical analysis

Temple doses to the doctor, scrub and scout nurse had a log-normal distribution, thus requiring a log-transformation for the analysis. Other variables such as fluoroscopy time, AK and DAP also exhibited a log-normal distribution based on the normal quantile plots. Results of log-transformed variables were reported as geometric means with 95% confidence intervals (CIs). STATA(15.1) and JMP Pro were used for all analyses, while Microsoft Excel was used for graphs.

## Results

**Fig. 2:**
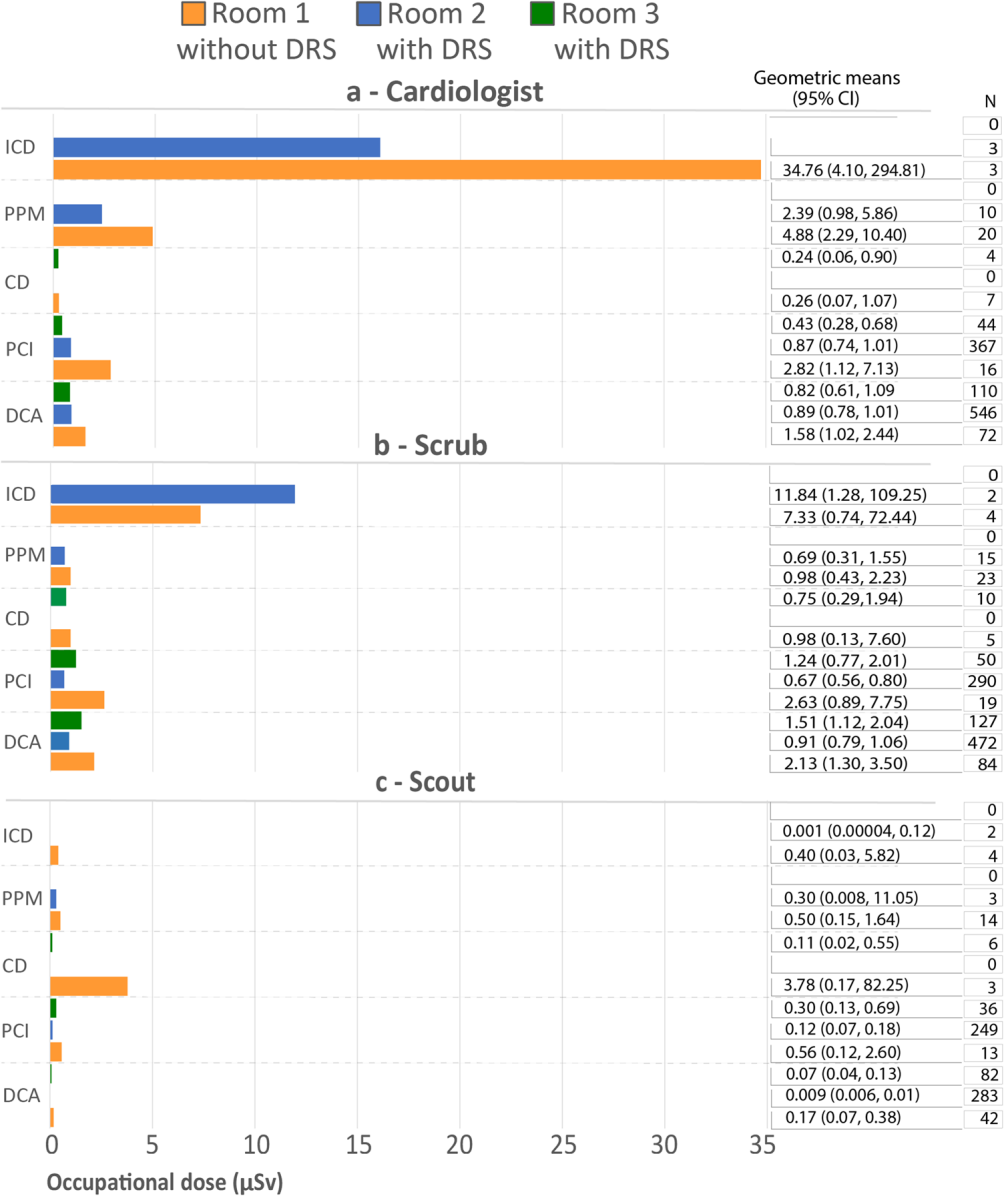
Occupational dose for the cardiologist (**a**), scrub nurse (**b**) and scout nurse (**c**) during diagnostic coronary angiograms (DCA), percutaneous coronary intervention (PCI), closure devices (CD), permanent pacemaker insertions (PPM) and implantable cardioverter defibrillator (ICD)

**Fig. 3:**
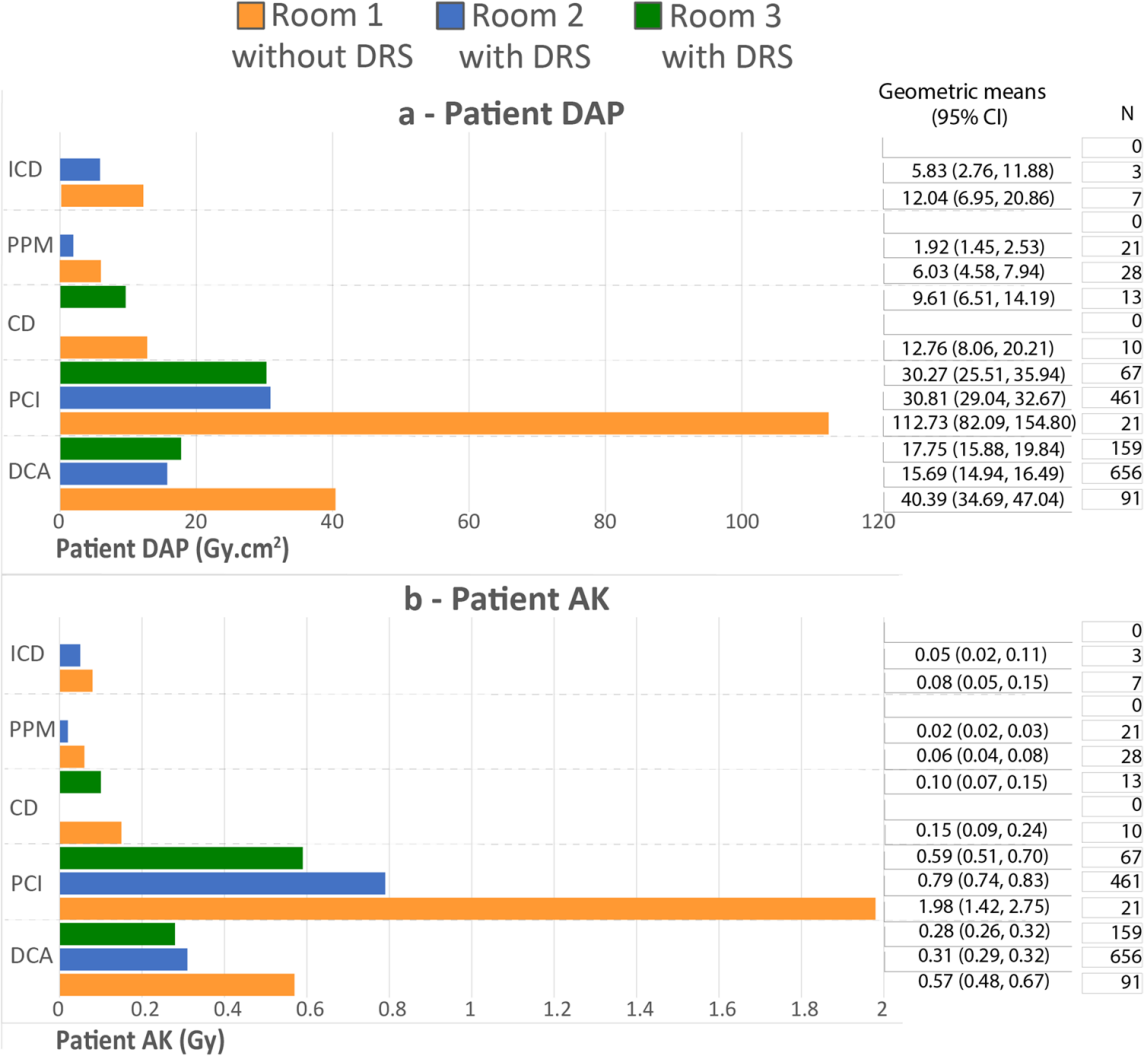
Patient dose area product (DAP) (**a**) and air kerma (AK)(**b**) during diagnostic coronary angiograms (DCA), percutaneous coronary intervention (PCI), closure devices (CD), permanent pacemaker insertions (PPM) and implantable cardioverter defibrillator (ICD)

Staff (Fig. 2) and patient (Fig. 3) dose measurements were collected in R1 (n = 157), R2 (n = 1141) and R3 (n = 239). There were no closure device procedures performed in R2 and no implantations of permanent pacemakers or cardioverter defibrillators were performed in R3.

As demonstrated in Fig. 2, the mean dose to staff was higher in R1 (without the DRS) in all categories, except scrub dose during implantation of BiV cardioverter defibrillators. The average dose to the cardiologist was higher in R1 during diagnostic coronary angiography (1.58 μSv), percutaneous coronary intervention (2.82 μSv), permanent pacemaker insertion (4.88 μSv) and implantation of cardioverter defibrillators (34.76 μSv) and was associated with a significantly higher dose compared to R2 and R3 during diagnostic coronary angiography (0.89/0.82 μSv) and percutaneous coronary intervention (0.87/0.43 μSv), as demonstrated by non-overlapping CIs. The mean dose to the scrub nurses was found to be higher during diagnostic coronary angiography (2.13 μSv), percutaneous coronary intervention (2.63 μSv), closure devices (0.98 μSv) and insertion of permanent pacemakers (0.75 μSv) in R1 and was significantly higher for diagnostic coronary angiography (0.91 μSv) and percutaneous coronary intervention (0.67 μSv) when compared with doses in R2. Average scout nurse dose in R1 was higher across all procedural categories and was related to significantly increased dose during diagnostic coronary angiography when compared with R2 (0.17/0.009 μSv, respectively).

The cardiologist had a higher average dose during closure devices in R1 (34.76 μSv) and R2 (16.06 μSv) compared with the scrub nurse (7.33/11.84 μSv for R1 and R2, respectively). The average dose was also higher for the cardiologist during the insertion of permanent pacemakers in R1 (4.88 μSv) and R2 (2.39 μSv) compared to the scrub nurse (0.98/0.69 μSv). Conversely, the average scrub nurse dose was found to be similar to that of the cardiologist during percutaneous coronary interventions (2.63/2.82 μSv in R1 for the nurse and cardiologist, respectively) and higher during diagnostic coronary angiography (1.51/0.82 μSv in R3), and closure devices (0.98/0.26 μSv in R1).

Average patient AK and DAP were higher in R1 during all included procedures (Fig. 3). AK in R1 was significantly higher than R2 and R3 during diagnostic coronary angiography (0.57/0.31/0.28 Gy for R1/R2/R3, respectively) and percutaneous coronary intervention (1.98/0.79/0.59 Gy). DAP was also significantly higher in R1 when compared to R2 and R3 during diagnostic coronary angiography (40.39/15.69/17.75 Gy·cm^2^) and percutaneous coronary intervention (1.98/0.79/0.59 Gy·cm^2^). In addition, R1 was related to significantly higher mean DAP (6.03 Gy·cm^2^) and AK (0.06 Gy) during permanent pacemaker procedures, with the mean dose in R2 (DAP—1.92 Gy·cm^2^/AK—0.02 Gy) being approximately one-third of that in R1.

When considering the entire dataset (regardless of the room), diagnostic coronary angiography and percutaneous coronary intervention procedures were associated with significantly higher patient AK and DAP compared to closure devices, permanent pacemaker and cardioverter defibrillator implantations. Additionally (when considering the entire dataset), the average occupational dose to the cardiologists was higher during implantation of permanent pacemakers and cardioverter defibrillators compared to diagnostic coronary angiography and percutaneous coronary interventions.

A comparison of fluoroscopy time, number of cine runs, contrast volume and patient body mass index (BMI) for each room was performed (Table 2). Males constituted 70% of patients, and the average BMI was similar for males (29.7) and females (29.6). Mean values of fluoroscopy time, number of cine runs, and patient BMI were comparable, with the only significant difference being that permanent pacemakers implanted in R2 were associated with less fluoroscopy time (2.31 min) when compared with R1 (6.66 min). There was a disparity between the BMI of patients having permanent pacemaker implantations in R1 and R2, as well as percutaneous coronary intervention in each of the rooms, but these differences did not reach statistical significance. A variation in mean fluoroscopy time during closure devices was also noted between R1 (8.93 min) and R2 (3.85 min), but this difference did not reach statistical significance.

**Table 2:** Geometric mean (95% CI) procedural parameters [mean for Pt BMI (95% CI)] for differing procedural variables per room

Room	Procedure	n	Fluoroscopy time (mins)	Cine runs^a^	Contrast (mls)	Patient BMI
1	ICD	7	8.93 (5.23, 15.22)	1.36 (0.37, 4.99)	0.09 (0.02, 0.42)	30.24 (24.99, 35.50)
2	ICD	3	3.85 (1.99, 7.47)	3.30 (1.75, 6.22)	0.03 (0.02, 0.06)	29.76 (22.68, 36.84)
3	ICD	0	–	–	–	–
1	PPM	28	6.66 (5.10, 8.70)	0.51 (0.27, 0.98)	0.007 (0.003, 0.015)	30.45 (27.78, 33.13)
2	PPM	21	2.31 (1,80, 2.96)	0.28 (0.22, 0.36)	0.002 (0.001, 0.002)	26.46 (23.72, 29.20)
3	PPM	0	–	–	–	–
1	CD	10	7.45 (4.76, 11.64)	11.80 (3.98,35.02)	33.73 (9.61, 118.37)	29.41 (25.01, 33.80)
2	CD	0	–	–	–	–
3	CD	13	6.82 (4.89, 9.50)	8.87 (7.30, 10.79)	31.57 (24.60, 40.53)	26.02 (22.37, 29.67)
1	PCI	21	11.43 (8.40, 15.55)	23.24 (10.97, 49.23)	162.75 (68.43, 387.06)	31.65 (28.62, 34.69)
2	PCI	461	11.29 (10.79, 11.91)	21.56 (20.49, 22.69)	179.51 (170.48, 189.02)	26.46 (23.72, 29.20)
3	PCI	67	11.61 (10.03, 13.43)	19.58 (17.96, 21.34)	154.56 (138.46, 172.53)	27.66 (26.05, 29.27)
1	DCA	91	2.70 (2.33, 3.13)	9.23 (6.43, 13.23)	73.56 (48.52, 111.53)	28.74 (27.28, 30.20)
2	DCA	656	3.08 (2.95, 3.22)	9.60 (9.20, 10.02)	82.03 (78.56, 85.66)	29.43 (28.95, 29.91)
3	DCA	159	3.09 (2.81, 3.40)	9.41 (8.90, 9.95)	84.37 (78.56, 90.62)	29.15 (28.11, 30.20)

*BMI* body mass index, *mins* minutes, *mls* millilitres, *CD* closure devices, *DCA* diagnostic coronary angiography, *ICD* implantable cardioverter defibrillator, *PCI* percutaneous coronary angiography, *PPM* permanent pacemaker

^a^Average number of cine acquisitions per procedure

## Discussion

It has been demonstrated that almost all procedures performed in the room without the ClarityIQ DRS (R1) had higher average staff and patient dose when compared to the measurements collected in the two rooms fitted with the DRS (R2 and R3).

If the quality of images produced by equipment with DRS installed was insufficient to visualize pathology, it would be expected that this would be reflected by a rise in the number of cine acquisitions, additional fluoroscopy time, and an increase in contrast volume. As demonstrated by the overlapping CIs (with the exception of permanent pacemaker insertions in R1 and R2), this study demonstrated no significant difference in contrast volume, fluoroscopy time, or the number of cine acquisitions between rooms with DRS (R2 and R3) and without (R1). Noting that the range of patient BMI was also similar across all rooms and procedures, this would indicate that equipment with DRS installed provided images of sufficient quality in the study center, which has been previously reported [22–24]. The average fluoroscopy time was significantly longer for permanent pacemaker procecedures performed in R1, which was determined to be due to the recent adoption of His bundle pacing in the department. Due to the smaller potential target area for lead placement, the procedure is technically challenging and leads to longer fluoroscopy times when compared with traditional right ventricular pacing [25].

Previous investigations favoured reporting the dose value of DAP, also termed kermaarea product (KAP), or air kerma-area product (P_KA_) [26] which provides a general dose metric for the total dose absorbed by tissue and reflects the risk of stochastic (cancer and genetic) effects [27]. The reduction in DAP from this study ranged from 25 to 73% (Table 3), which mirrors previous findings (Table 1).

**Table 3:** Percentage mean dose reduction for patients and staff in R2 and R3 compared with R1 (without DRS)

Procedure	Room	Cardiologist (% dose reduction)	n	Scrub (% dose reduction)	n	Scout (% dose reduction)	n	Patient AK (% dose reduction)	n	Patient DAP (% dose reduction)	n
ICD	2	54	3	+ 38	2	99	2	27	3	52	3
PPM	2	51	10	30	15	40	3	33	21	68	21
CD	3	8	4	33	10	97	6	33	13	25	13
PCI	2	69	367	75	290	79	249	60	461	73	461
PCI	3	85	44	53	50	46	36	70	67	73	67
DCA	2	44	546	57	472	95	283	46	656	61	656
DCA	3	48	110	29	127	59	82	51	159	56	159

*AK* air kerma, *DAP* dose area product, *CD* closure devices, *DCA* diagnostic coronary angiography, *ICD* implantable cardioverter defibrillator, *n* number of procedures, *PCI* percutaneous coronary angiography. *PPM* permanent pacemaker, *R1* room1, *R2* room 2, *R3* room 3

This study found similar levels of reduction in patient DAP of 56–61% during diagnostic coronary angiography as Eloot et al. (75%), Balter et al. (34–44%), ten Cate et al. (53%), Kastrati et al. (65%) and Kraemer et al. (56%) [23, 28–31]. Reductions in DAP of 69% during percutaneous coronary interventions were also recorded by Kastrati et al. [30], which is comparable to the 73% found in this investigation. Numerous authors outlined the reduction in dose during procedures to revascularize chronic total occlusions of the coronary arteries [13, 28, 32, 33]. There is not a high volume of treatments undertaken for chronic total occlusions at the center in which this study was undertaken, so these procedures were included in the percutaneous coronary intervention data. Comparison of dose rates specifically for chronic total occlusion procedures are thus beyond the scope of this study. The percentage reduction reported by Hoffman et al. of 60% during insertion of permanent pacemakers and implantable cardioverter defibrillators were similar to the 52–68% found in this study [15].

In addition to DAP, the value of AK, also referred to as reference, incident or cumulative air kerma (K_a,r_), was collected as a component of this study. AK provides a rough estimate of peak skin dose and assists in predicting potential tissue reactions, or deterministic effects. The primary concern regarding radiation to the patient during fluoroscopically guided procedures are tissue reactions such as skin erythema, and hence AK is the preferred dose indicator [34]. R1 was associated with larger average AK during all procedures and was statistically higher during diagnostic coronary angiography, percutaneous coronary intervention and permanent pacemaker procedures.

The effect of DRS on patient and staff dose during implantation of closure devices in adults has been sparsely investigated, with authors predominantly focusing on dose comparisons for patients[35] or as a phantom study [36]. Sharma et al. reported a 69% reduction in AK during deployment of atrial closure devices, which is higher than the 33% found in our study [35]. This may be due to the inclusion of ASD and PFO closure procedures in the closure devices dataset in this study. It is noteworthy that the reduction in AK for the insertion of permanent pacemakers and implantable cardioverter defibrillators of 27–33% was also lower than Sharma et al.’s value of 96%, but there was a marked difference in the median reported dose in the lab without ClarityIQ for the insertion of permanent pacemakers/implantable cardioverter defibrillators of 87 mGy, compared to a mean of 6-8 mGy in the current study, and this may indicate that the base patient exposures were higher and explain the greater dose reduction. Our study has demonstrated a reduction in DAP during insertion of closure devices of 25%, and although there is currently no information on adults to compare with, Sullivan et al. reported a 68% reduction for device occlusions of atrial and ventricular septal defects, patent ductus arterioses, and venous and arterial collaterals on paediatric patients [8].

There is a notable lack of research investigating differences in occupational exposure after the installation of ClarityIQ during cardiac procedures, especially to nursing staff. Any reduction in patient dose is associated with a decrease in the radiation scatter impinging on staff [37, 38], and this is reflected in the reduction in staff dose across almost all procedures and staff roles. One notable exception is the increased scrub nurse dose demonstrated during implantable cardioverter defibrillators in R2. Further examination identified a single case was responsible for the unexpectedly high average dose measurement. The dose to the scrub nurse during this case was one of the highest recorded for all procedures included in the study at 61 μSv (R2 implantable cardioverter defibrillator average—31 μSv, full dataset average—2.7 μSv), and also resulted in a relatively high cardiologist dose of 36 μSv (R2 implantable cardioverter defibrillator average—19 μSv, full dataset average—3 μSv). Patient AK and DAP were also higher than the other implantable cardioverter defibrillators performed in R2. As the patient had a lower BMI (25) compared with the average for implantable cardioverter defibrillators (30), the increased dose to the patient, cardiologist and scrub nurse was concluded to be due to procedural complexity. This discrepancy in average dose value was also due to the small number of implantable cardioverter defibrillator procedures included in the sample (n = 10).

Our study has demonstrated a decrease in staff dose when using DRS, which corresponds to studies reporting a reduction of cardiologist dose of 50% for ablations procedures [9] and a 60% decrease in operator dose during endovascular procedures [10]. However, the results contrast with other studies investigating the effect of DRS on occupational dose. Salinas et al. reported a 36% reduction in DAP during chronic total occlusion intervention, but also noted no reduction in occupational dose and, in fact, an increase in scatter dose [13]. This was also found by Sanchez et al. in a study comparing dose in over 5000 procedures with the explanation that, while there is a reduction in radiation, due to the additional filtration, there was an increase in the average energy of the photons in the primary beam and an associated increase in scattered radiation [14]. Investigations of the effect of DRS during endovascular procedures have demonstrated a reduction in operator dose [10], as well as other in-room staff [11, 12]. Additional studies investigating the effect of DRS on staff (including non-operator) dose during cardiac procedures are required.

This study has demonstrated that while implantation of permanent pacemaker and cardioverter defibrillator procedures result in less patient dose than diagnostic coronary angiography, percutaneous coronary intervention and the deployment of cardiac closure devices, the cardiologist is exposed to higher levels of temple exposure. This can be explained by the reluctance of the cardiologists to utilize the ceiling-mounted lead shield when situated on the patient’s left side (Fig. 1B). Ideally, room design should include a movable ceiling-mounted lead shield on the left side of the patient table to allow for easy positioning and use during implantation of permanent pacemakers and cardioverter defibrillators. It is also worth noting that while most contemporary literature reports that operator dose during fluoroscopically guided procedures is higher than that of other in-room staff, our study has demonstrated that the mean dose to the scrub nurse was higher than the cardiologist during diagnostic coronary angiography, insertion of closure devices, and similar during percutaneous coronary intervention procedures as demonstrated in Table 4. The average scrub nurse dose for diagnostic coronary angiography was 2.13/0.91/1.51 μSv for R1, R2 and R3 respectively, compared to the average dose to the cardiologist of 1.58/0.89/0.82 μSv. One possible explanation for the higher dose to the scrub nurse is the positioning of the ceiling-mounted lead shield during procedures. It has been previously reported that if the movable lead shield is located directly in front of the fluoroscopic operator, it may afford protection only to that person. Hence, although the scrub nurse is positioned further away from the maximum area of scattered radiation, the absence of a physical barrier may result in a higher dose than the operator[39]. Another possible reason for increased dose during diagnostic coronary angiography is the inclusion of cases performed by non-interventional cardiologists, who may be less aware of radiation minimization strategies, leading to increased patient and staff dose. It should be noted that this is not supported by any significant increase in the patient dose data for procedures performed by non-interventionalists in this study.

**Table 4:** Geometric mean (95% CI) for cardiologist and scrub nurse during closure devices, percutaneous coronary interventions and diagnostic coronary angiogram

Procedure	Room	Cardiologist (μSv)	Scrub nurse (μSv)
CD	1	0.26	0.98
	3	0.24	0.75
PCI	1	2.82	2.63
	2	0.87	0.67
	3	0.43	1.24
DCA	1	1.58	2.13
	2	0.89	0.91
	3	0.82	1.51

*CD* closure device, *DCA* diagnostic coronary angiography, *PCI* percutaneous coronary angiography, *μSv* microSievert

The occupational dose during implantation of closure devices had surprising results with the levels of average dose highest to the scout nurse (3.78 μSv), followed by the scrub nurse (0.98 μSv) and lowest for the cardiologist (0.26 μSv). The authors found this difficult to explain but note that the results may have been influenced by the limited number (< 8 cases in each staff category) of closure device cases included in the dataset. This may be a topic for further investigation.

This study has shown that the dose to the nursing staff may exceed that of the cardiologist. The potential for occupational exposure in a cath lab varies with cardiologist preference, departmental protocols, the complexity and location of cardiac pathology, x-ray tube angle and availability and use of personal protective equipment. The results of this study should encourage and motivate cath lab staff and managers to investigate and compare dose levels within their specific settings to identify procedures that may increase the dose to staff. Numerous authors have reported that the use of ClarityIQ results in little or no perceivable loss of image quality [6, 12, 24, 29, 31, 40–42]. While this manuscript highlights procedures performed on Philips’s systems exclusively, Gislason-Lee et al. report a similar (48–61%) reduction in patient dose when systems with ClarityIQ software is compared to Siemens Axion Artis x-ray machine. Significant reductions in patient exposure (48–77%) with comparable image quality have also been shown when comparing later generations of imaging technology from Siemen systems[43–45].

### Limitations

The main limitation of this study was the single-center design which may limit the generalizability of our results. Variations in practice may result in either an over or underestimation of the effectiveness of DRS within local settings. An assessment of image quality was not included in our study. The number of cine runs and fluoroscopy time were similar in the rooms with DRS compared to the room without, indicating that additional fluoroscopy and cine runs were not required to produce images of sufficient quality. Furthermore, favourable comparison of image quality with DRS has been well reported previously.

Another limitation is the small sample size of closure device procedures, permanent pacemaker and cardioverter defibrillator implantations in specific rooms which would have influenced the reliability of our results.

## Conclusions

Patient and staff dose during fluoroscopically guided cardiac procedures could be reduced by approximately 50% when performed with equipment installed with ClarityIQ DRS. Administrators should ensure timely upgrades to angiographic equipment to safeguard patients and staff against the potentially adverse effects of radiation exposure and adhere to the principle of keeping the dose as low as reasonably achievable. There is currently contradictory literature regarding the reduction in staff dose as a result of implementing noise reduction technology, and while this study has demonstrated a convincing decrease in dose to the cardiologist and nursing staff, additional research is recommended. Nursing staff should also be aware that their levels of radiation dose during cardiac procedures may come close to or even exceed that of the cardiologist.

## Data Availability

The data that support the findings of this study are openly available at https://researchdatafinder.qut.edu.au/display/n8649; 10.25912/RDF_1621382081526.
